# Continuous infusion OPAT via elastomeric pumps: effectiveness, safety, and cost-saving potential in a real-world Italian cohort

**DOI:** 10.1007/s15010-025-02671-0

**Published:** 2025-10-31

**Authors:** Stella Babich, Stefano Di Bella, Raffaele De Rivo, Oyewole Christopher Durojaiye, Antonio Lovecchio, Andrea Misin, Madalina Straciug, Ylenia Gobbo, Angela Dellaluce, Michela Palmolungo, Massimiliano Fabricci, Filippo Giorgio Di Girolamo, Chiara Roni, Jacopo Monticelli

**Affiliations:** 1 Infectious Disease Unit, Trieste University Hospital, Piazza dell’Ospitale 1, Trieste, 34125 Italy; 2 Clinical Department of Medical, Surgical, and Health Sciences, Trieste University, Trieste, Italy; 3 School of Medicine, University of Trieste, Trieste, Italy; 4 Department of Infection and Tropical Medicine, Sheffield Teaching Hospitals NHS Foundation Trust, Sheffield, UK; 5 Department of Microbiology, University Hospitals of Derby and Burton NHS Foundation Trust, Derby, UK; 6 General Directorate, Trieste University Hospital (ASUGI), Trieste, Italy; 7 Medical Directorate, Trieste University Hospital (ASUGI), Trieste, Italy; 8 Hospital Pharmacy, Cattinara Hospital, Azienda Sanitaria Universitaria Giuliano Isontina, Trieste, Italy

**Keywords:** OPAT, Elastomeric pumps, Continuous infusion, Antimicrobial stewardship, Cost-analysis

## Abstract

**Purpose:**

To evaluate clinical outcomes, safety, patient-reported satisfaction, and cost-effectiveness of elastomeric pump-based Outpatient Parenteral Antimicrobial Therapy (OPAT) over six years at an Italian tertiary center.

**Methods:**

This retrospective single-center study included 76 adult patients treated with continuous-infusion OPAT via elastomeric pumps between 2019 and 2024 at the University Hospital of Trieste, Italy.

**Results:**

A total of 1,934 elastomeric pump-based OPAT days were delivered (median duration of 22.9 days). Clinical cure was achieved in 85.5% of patients; recurrence and failure occurred in 6.2% and 7.9%, respectively. Most frequent indications were skin/soft tissue and surgical site infections (25.9%), complicated urinary tract infections (22.4%), and bone/joint infections (16.4%). Pathogens were mainly Gram-negative (70.7%), including *Enterobacterales* (40.2%, 57.6% ESBL-producing), *Pseudomonas aeruginosa* (26.8%), and *Staphylococcus aureus* (17.1%, 28.6% methicillin-resistant *S. aureus*). The most used antibiotics were piperacillin/tazobactam (51.3%), cefepime (12.5%) and ceftolozane/tazobactam (7.5%). Adverse events were observed in 13.75% of treatments, primarily vascular access-related (5.7 events/1,000 OPAT-days); drug-related adverse events occurred in 7.8% of patients (3.1 events/1,000 OPAT-days). Among contacted patients (75% response rate), 83.7% expressed willingness to reuse the pump. Total OPAT costs were €62,190.64 compared to an estimated €773,600.00 for inpatient care, yielding a 92% cost reduction (€711,409 saved).

**Conclusion:**

Elastomeric pump-based OPAT is a clinically effective, well-tolerated, and economically advantageous option for selected infections. Its integration into stewardship programs supports broader implementation within modern, sustainable infectious disease care models.

## Introduction

The increasing demand for cost-effective and patient-centered healthcare has driven the global expansion of Outpatient Parenteral Antimicrobial Therapy (OPAT) programs. This approach enables patients requiring prolonged intravenous antibiotic treatment to receive therapies outside of a hospital setting, improving their quality of life while reducing healthcare-associated costs and risks, such as nosocomial infections [1]. This approach also supports early discharge and admission avoidance, allowing patients to resume work, school, and social duties. Among the various drug delivery systems employed in OPAT, elastomeric pumps have emerged as a valuable option due to their portability, ease of use, and ability to provide continuous drug infusion without the requiring external power sources [2]. These characteristics make elastomeric devices particularly well-suited for time-dependent antibiotics, as they maintain therapeutic drug levels over prolonged periods, thus enhancing pharmacodynamic efficacy [3]. However, their use requires careful patient selection and close monitoring to ensure safety and efficacy [4–6].

Beyond its logistical advantages, OPAT also contributes to antimicrobial stewardship, particularly in the treatment of infections caused by extended-spectrum beta-lactamase (ESBL)-producing pathogens. The use of continuous-infusion beta-lactam antibiotics via elastomeric pumps facilitates carbapenem-sparing regimens, reducing unnecessary exposure to carbapenems and mitigating the risk of selecting for carbapenem-resistant *Enterobacterales* (CRE) [7]. Moreover, recent studies indicate that OPAT should be systematically integrated into antimicrobial stewardship programs, ensuring that treatment regimens align with evidence-based guidelines and minimizing resistance selection pressures [8].

Despite the robust evidence supporting OPAT, its implementation remains heterogeneous across healthcare systems. While some countries have embraced community-based models that empower patients or caregivers to administer therapy independently, others rely primarily on hospital-supervised administration [9]. In Italy, the use of elastomeric pumps in OPAT has gradually expanded, reflecting increasing awareness of their clinical and economic benefits. Nevertheless, their adoption is still limited to specific institutions, suggesting significant potential for broader integration into wider national healthcare settings.

Furthermore, economic and environmental considerations are additional factors supporting the adoption of OPAT. Studies report reductions in healthcare expenditures ranging from 50% to 75% compared to inpatient treatment, attributed to decreased hospital stays, lower personnel costs, and optimized antibiotic selection [10–17]. Cost-effectiveness evaluations suggest that the financial benefits of OPAT are maximized when combined with rigorous patient monitoring and optimized selection of antimicrobial agents [18].

In addition, recent research demonstrates that OPAT significantly reduces environmental impact compared to inpatient intravenous antibiotic therapy [19]. Self-administered OPAT is associated with up to 85% lower CO_2_ emissions, 78% less water consumption, and a 91% reduction in waste generation, making it a more sustainable approach to healthcare delivery [19].

To contribute to the existing body of evidence, we conducted a retrospective observational study evaluating our six-year experience with elastomeric pumps in the OPAT program at our center in Trieste, Italy. The study aims to provide insights into clinical outcomes, safety profile, and the economic impact of this approach.

## Materials and methods

### Study design and outcomes assessed

This retrospective, observational, single-center study was conducted at the Infectious Diseases Unit of Trieste University Hospital between January 2019 and December 2024. The primary outcome was to provide a descriptive overview of our six-year experience with elastomeric pumps in the context of OPAT, focusing on clinical outcomes and safety profiles. Secondary outcomes included recurrence rates, device tolerability, and a comparative cost analysis between outpatient and inpatient care.

### Definition

*Clinical cure* was defined as the complete resolution of infection-related signs and symptoms without the need for further antimicrobial therapy at the end of treatment. This aligned with the therapeutic goal of cure, successful completion of the planned OPAT course with no indication for prolonged antimicrobial use.

*Recurrence* was referred to the reappearance of clinical signs and symptoms of infection, with or without microbiological confirmation, within 90 days of treatment completion.

*Treatment failure* included any case requiring modification of the antimicrobial regimen due to clinical deterioration, hospital readmission for the same infection, or documented disease progression despite ongoing therapy.

*Improvement* described cases in which the planned OPAT course was completed within a broader infection management strategy, e.g., when subsequent surgical intervention was anticipated, long-term oral suppressive therapy was required, or potentially infected prosthetic material remained in situ.

*Palliation* applied to patients with significant comorbidities and established ceilings of care, where OPAT was initiated with non-curative intent and death was considered a foreseeable outcome.

Adverse events were categorized as either drug-related or vascular access-related (e.g., thrombosis, infection, occlusion, catheter malfunction). Drug-related events were further stratified by severity as mild (e.g., gastrointestinal intolerance, localized urticaria), moderate (e.g., red man syndrome, thrombocytopenia > 50,000/mm³, leukopenia > 1,000/mm³), or severe (e.g., hypersensitivity reactions requiring treatment discontinuation, thrombocytopenia < 50,000/mm³, leukopenia < 1,000/mm³).

### Inclusion and exclusion criteria

Patients were selected based on predefined eligibility criteria to ensure suitability for OPAT [4–6, 8, 20–23]. The study population included adults (aged ≥ 18 years) with active infections requiring intravenous antibiotic therapy and managed with elastomeric pumps between 2019 and 2024.

Inclusion criteria required patients to be clinically and hemodynamically stable, have reliable deep vascular catheter, such as Midline or Peripherally Inserted Central Catheter (PICC), and lack effective oral antibiotic alternatives. Additionally, eligible patients needed a suitable home or outpatient environment that supported safe OPAT administration, with adequate caregiver support and daily access to the outpatient clinic.

Exclusion criteria included the need for continued inpatient care, medical instability, or social and clinical conditions that could compromise adherence or safety.

### OPAT protocol and pump management

At our institution, OPAT delivery is coordinated through a dedicated multidisciplinary OPAT service composed of the infectious disease specialist overseeing patient care, clinical pharmacists managing drug procurement, trained nurses responsible for drug preparation and administration, and a specialized vascular access team for the placement and maintenance of Midline and PICC lines. Additional consultants (e.g., orthopaedic surgeons, urologists, or other specialists) are involved as needed, depending on the infection type and clinical situation, to provide specific expertise or source control when required.

Antibiotics were administered using the Baxter INFUSOR™ LV elastomeric pumps at a fixed infusion rate of 10 mL/h (product code 2C1063KP). Antimicrobial selection was guided by established stability data from the literature and validated protocols, ensuring compatibility with the device under defined conditions of preparation, storage, and administration [3, 24–33].

Pumps were prepared onsite and connected by trained healthcare professionals during daily outpatient visits. Patients attended the clinic each day for infusion, where the elastomeric pump was replaced and therapy administered. Follow-up evaluations included physical examinations and laboratory testing, scheduled according to the clinical judgment of the treating physician. Vascular catheters (Midline or PICC) were managed through weekly dressing changes and were assessed daily for signs of infection, occlusion, or malfunction. All adverse events, including vascular access complications, antibiotic-related reactions, or pump malfunctions, were systematically recorded, and in case of any such issues, patients had prioritized access to the Infectious Diseases Unit for prompt evaluation and management. In addition, patients underwent periodic clinical reassessments and, when appropriate, further diagnostic investigations as needed to evaluate treatment response and guide antimicrobial stewardship decisions such as de-escalation when feasible.

**Fig. 1:**
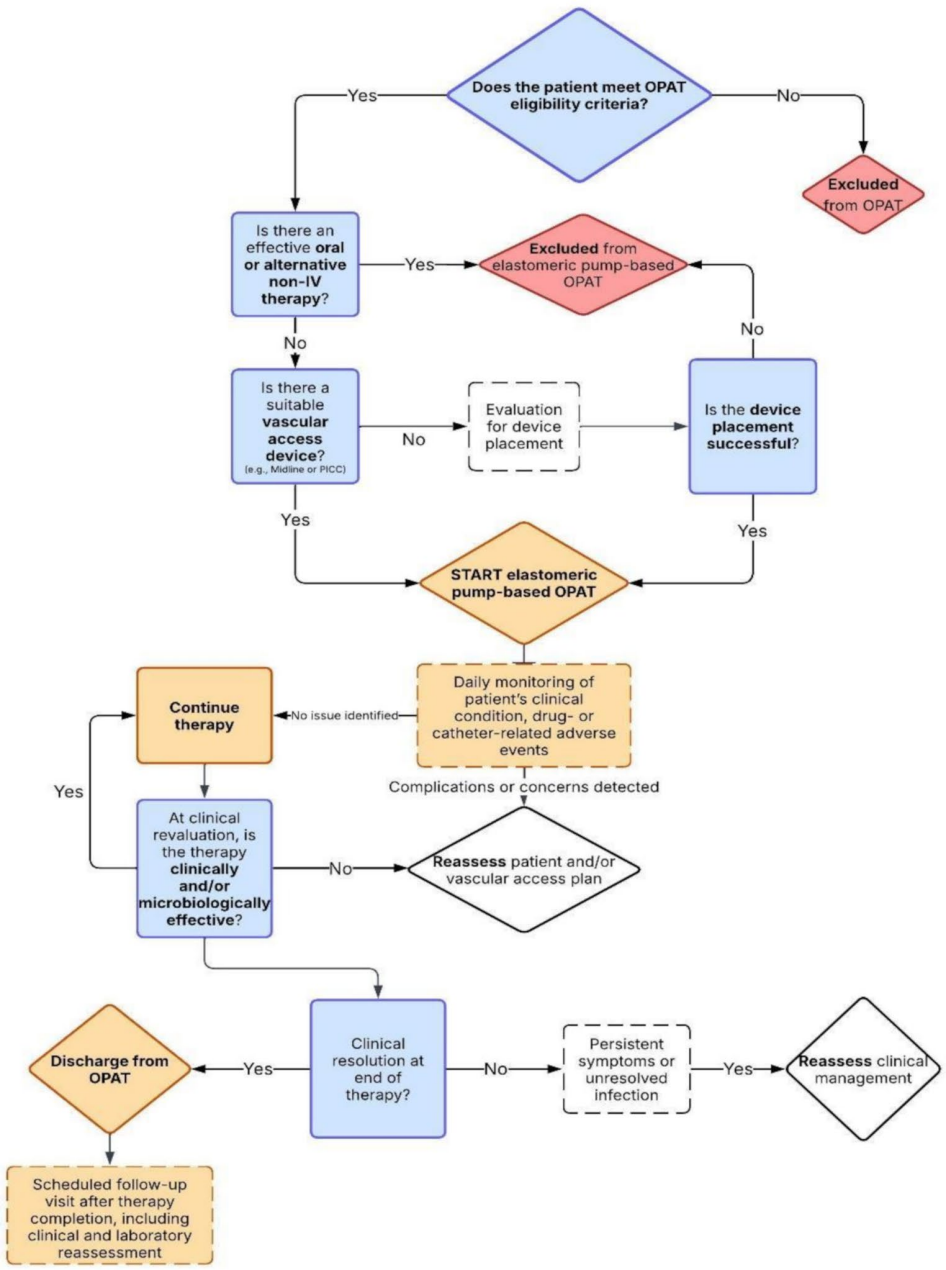
Flowchart of the patient management process for OPAT with elastomeric pumps in the Infectious Diseases Department

The operational workflow for patient selection and management within the elastomeric pump-based OPAT program is summarized in Fig. 1.

Most patients received treatment under a day hospital regimen; others continued OPAT after hospital discharge. To enhance comprehension and adherence, patients were provided with an informational leaflet covering device function and frequently asked questions regarding elastomeric pump systems.

### Data collection

Data were prospectively recorded in a dedicated database, including demographic variables (age, sex), clinical diagnoses (infection type and severity), microbiological findings (pathogens, resistance profile), and treatment specifics (antibiotic used, treatment duration, dose, frequency, and combination regimens). Additional variables included adverse events (drug-related side effects and vascular access complications), and clinical outcomes.

### Patient tolerability assessment

To assess patient experience, all study participants were contacted for a structured telephone interview in March 2025. A standardized questionnaire, adapted from the Comparing Home Infusion Devices (CHID) study, was used to assess usability, tolerability, comfort, impact on daily life, and overall satisfaction with elastomeric pump therapy [34] (the full questionnaire is available in the **Supplementary Materials**). Responses were analysed to explore patient-reported outcomes and preferences regarding elastomeric pump-based OPAT.

### Cost analysis

A cost-minimization analysis was conducted to evaluate the direct medical costs associated with OPAT using elastomeric pumps compared to conventional inpatient intravenous therapy. The analysis was carried out from the perspective of the Italian National Health Service (public payer), over the period 2019–2024. Only direct healthcare costs sustained by the hospital were considered, and the time horizon was limited to the duration of each intravenous treatment episode.

Unit costs for OPAT included elastomeric pumps (9.96€ per unit per day), nurse time for daily 30-minute administration and blood sampling at the outpatient clinic (11€), outpatient clinic operational overheads (3€ per 30-minute visit) and infectious disease consultations (29€ for the initial visit, 28€ per 30-minute weekly follow-up). Vascular access placement (Midline or PICC) was estimated at 74 € per procedure (60 € for the catheter, 11 € for 30-minutes nurse time, and 3 € for clinic overhead related to the insertion procedure), assuming one deep venous access per patient. Daily inpatient care was cost at 400€, based on standardized tariffs for medium-intensity hospital wards, and included staff, accommodation, meals, and fixed overheads.

Costs of antimicrobial agents and routine laboratory testing were excluded, as they were assumed to be equivalent in both settings. Similarly, transportation costs were excluded as they were borne by patients and did not impact hospital expenditures. No discounting was applied, as all treatment episodes had a time horizon shorter than one year. All cost estimates were calculated using constant 2025 Euros (i.e.: year-specific, inflation-adjusted value at the time of writing), applied retrospectively to the entire study period for consistency.

No attempt was made to quantify quality-adjusted life years (QALYs), early return to work, or broader societal benefits. Likewise, the potential reduction in nosocomial infections associated with outpatient management was not assessed. The analysis was strictly limited to a comparison of the direct costs related to the mode of antimicrobial administration (inpatient vs. OPAT), assuming equivalent clinical efficacy.

### Ethical approval

This study was approved by the Institutional Review Board of the University Hospital of Trieste and conducted in accordance with the Declaration of Helsinki. Due to its retrospective nature, the requirement for informed consent was waived as per institutional and ethical guidelines.

## Results

Over the six-year study period, a total of 76 patients were treated with elastomeric pumps at our institution. There was a progressive increase in the number of treated patients, from 7 in 2019 to a peak of 26 in 2023, with a temporary reduction in admissions observed in 2021 due to the impact of the SARS-CoV-2 pandemic.

The most frequent infection sites were soft tissue infections (25.9%), followed by complicated urinary tract infections (22.4%). Baseline characteristics, OPAT indications, treatment details, and clinical outcomes are summarised in Table 1.

**Table 1:** Baseline characteristics, OPAT indications, treatment details, and clinical outcomes of the study population (*n* = 76)

Population	Total (*n* = 76)
**Age**, **median [IQR]**	56.1 [30.0]
**Male sex** ***n***, **(%)**	49 (64.5)
**CCI**, **median [IQR]**	3.12 [5.0]
**Indication for OPAT** ***n*. (%)**	
SSTI-SSI	22 (25.9)
cUTI	19 (22.4)
Bone and joint infections	14 (16.4)
cIAI	9 (10.6)
Respiratory infections	8 (9.4)
BSI	6 (7.1)
Central Nervous System Infection	4 (4.7)
Endocarditis and device-related infection	3 (3.5)
**Duration for OPAT with elastomeric pumps**, **median (days)**	22.9
**Transitioned from prior inpatient intravenous therapy (%)**	43.4%
**Day of intravenous therapy before transition**	11.1
**Type of access (%)**	
MidLine	88.75
PICC	5
Other	6.25
**Concomitant antimicrobial therapy (%)**	31.2
**Infection outcome** ***n*. (%)**	
Clinical cure	65 (85.5)
Clinical improvement	4 (5.3)
Treatment failure	6 (7.9)
Palliation intent	1 (1.3)
**Recurrence rate**, ***n*** **(%)**	4 (6.2)

Microbiological analysis revealed that 70.7% of the 82 isolates in patients receveing OPAT via elastomeric pump were Gram-negative bacteria. The most identified pathogens were *Enterobacterales* (40.2%), *Pseudomonas aeruginosa* (26.8%), and *Staphylococcus aureus* (17.1%). ESBL-producing *Enterobacterales* were detected in 57.6% of cases. Microbiological findings are detailed in Table 2.

**Table 2:** Pathogens identified in the study population (*n* = 82), including those isolated in polymicrobial infections (*n* = 15)

Pathogens	Total (*n* = 82) (%)	Isolates in Polymicrobial Infections (*n* = 15)
*Enterobacterales*	33 (40.2)	9
ESBL-producing *Enterobacterales*	19 (57.6% of *Enterobacterales* isolated)	
*Pseudomonas aeruginosa*	22 (26.8)	9
*Staphylococcus aureus* (MSSA)	10 (12.2)	4
*Staphylococcus aureus* (MRSA)	4 (4.9)	2
Other Gram-positive bacteria	5 (6.1)	3
Candida spp. [*C. albicans*, *C. kefyr (Kluyveromyces marxianus)*, *C. krusei (Pichia kudriavzevii*)]	4 (4.9)	2
Other Gram-negative bacteria	3 (3.7)	0
Viral pathogens	1 (1.2)	0

Clinical cure was achieved in 65 patients (85.5%), with 4 cases of recurrence (6.2%), 6 treatment failures (7.9%), 4 clinical improvements (5.3%), 1 case managed with palliative intent (1.3%).

**Fig. 2:**
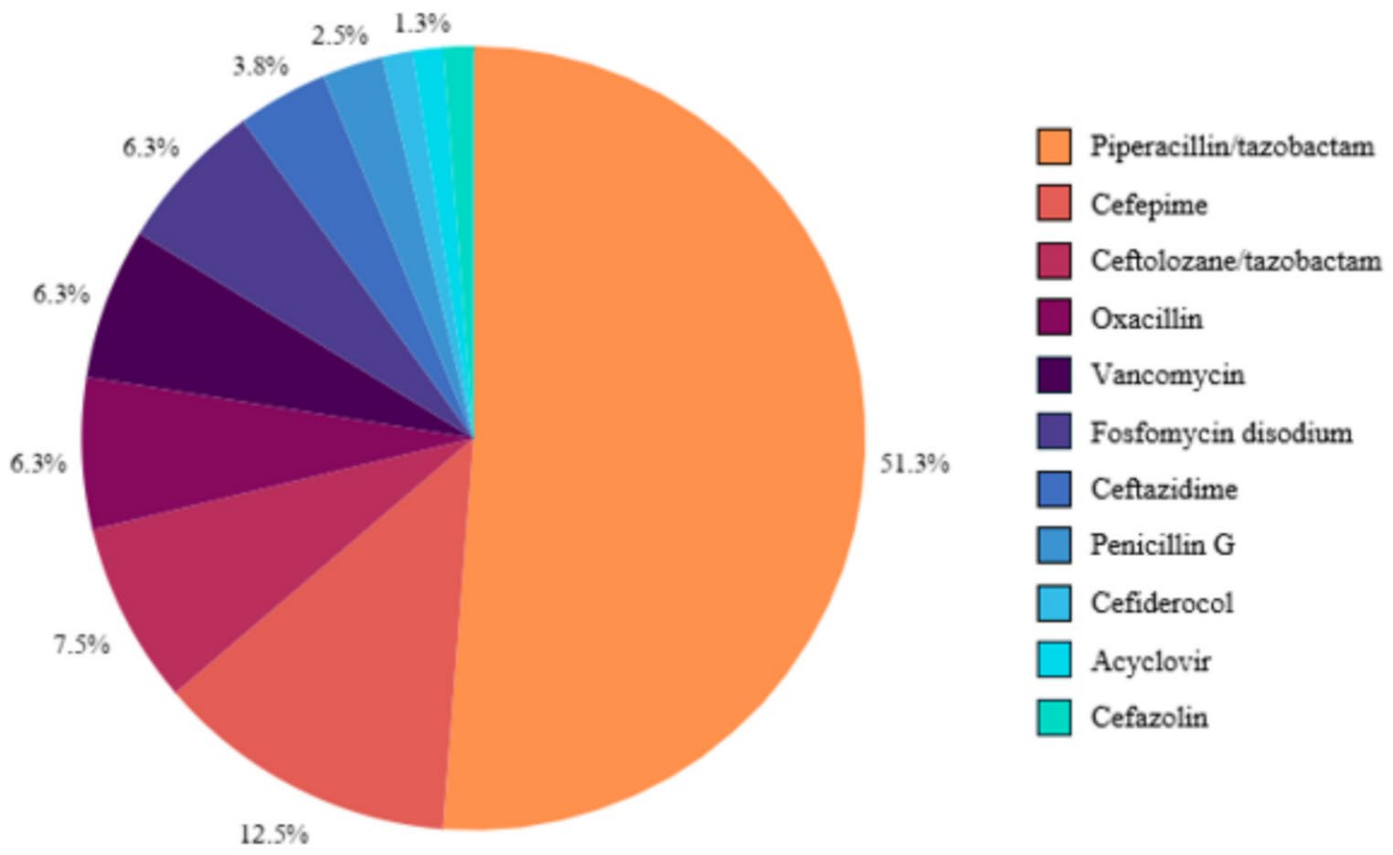
Prescriptions administered during OPAT (*n* = 80)

The most frequently prescribed antibiotics were piperacillin/tazobactam (51.25%), cefepime (12.5%), and ceftolozane/tazobactam (7.5%), followed by Fosfomycin disodium, oxacillin and vancomycin (each 6.25%). Other less common antibiotics included ceftazidime, penicillin G, cefazolin, cefiderocol. OPAT prescriptions are detailed in Fig. 2.

Combination therapy was employed in 31.2% of cases. The cumulative duration of therapy with elastomeric pumps was 1,934 days, with a mean duration of approximately 22.9 days per patient. Overall, 56.6% of patients initiated OPAT directly with elastomeric pumps, whereas 43.4% transitioned from prior inpatient intravenous therapy, with a median inpatient intravenous treatment duration of approximately 11.1 days before OPAT initiation. From an antimicrobial stewardship perspective, in 18 patients the use of piperacillin/tazobactam or ceftolozane/tazobactam, primarily for ESBL-producing *Enterobacterales*, allowed us to avoid prescribing carbapenems (ertapenem) in the OPAT setting, resulting in a total of 431 carbapenem treatment days spared.

Venous access devices included Midline catheters (88.75%), PICC (5.0%), central venous catheters (CVC, 1.25%), and peripheral venous catheters (5.0%). Among the 80 elastomeric pump treatments, 11 complications were reported in 10 patients (13.75% of treatments; 13.16% of patients), of which catheter-related thrombosis was the most frequent reported (27.3%). The estimated device-related complication rate was approximately 5.7 events per 1,000 therapy days. AEs and catheter-related complications are summarized in Table 3.

**Table 3:** Adverse events observed during OPAT with elastomeric pumps, stratified by type and severity, with incidence rates per 1,000 therapy days

Type of adverse event (AE)	*n* (%)
**AE Drug related** ***n*. (%)**	6 (7.8)
Mild	3 (50.0)
Moderate	2 (33.3)
Severe	1 (16.7)
AE drug related, *n*. events per 1000 OPAT-days	3.1
**AE Vascular-access related** ***n*. (%)**	**11 (13.75)**
Thrombosis	3 (27.3)
Superficial vein thrombosis	2 (18.2)
Deep vein thrombosis	1 (9.1)
Device inadequate emptying	2 (18.2)
Connector rupture	1 (9.1)
PICC fissuring	1 (9.1)
Irreversible occlusion	1 (9.1)
Suspected ulnar nerve injury requiring Midline replacement	1 (9.1)
Accidental self-removal	1 (9.1)
Pain at the Midline insertion site leading to removal	1 (9.1)
AE vascular-access related, *n*. events per 1000 OPAT-days	5.7
**Infection related to vascular-catheter** ***n*. (%)**	**2 (2.6)**
Bloodstream infection	1 (50)
Candidemia	1 (50)
Infection related to vascular catheter, *n*. events per 1000 OPAT-days	1.03

Pharmacological adverse events were reported in 7.8% of patients, predominantly mild reactions such as urticaria and gastrointestinal intolerance. Moderate reactions included “red man syndrome” and leukopenia, while severe reactions (1.37%) included thrombocytopenia. Only in 3 patients’ therapy had to be discontinued or switched to another molecule for a drug-related AEs. The estimated drug complication rate was 3.1 events per 1,000 therapy days.

Patient-reported outcomes were assessed through a structured telephone questionnaire conducted in March 2025. Among the 49 patients contacted (75% response rate), the majority had used the elastomeric pump for 1–4 weeks (53%), followed by those treated for more than 4 weeks (40%). The device was well tolerated, with over 95% rating its shape and noise level as “absolutely acceptable,” and over 65% doing so for weight. A small proportion (up to 10%) rated certain aspects as only “partially acceptable,” primarily concerning weight.

In terms of perceived safety, 71.4% of respondents reported feeling *very safe* using the device, with an additional 20.4% expressing agreement. Satisfaction with pump performance was high, with nearly 80% stating it met their expectations. Importantly, the willingness to reuse the device if needed was expressed by 83.7% of patients, underscoring its usability and acceptability in real-world outpatient care. Statistical analysis of the questionnaire responses confirmed high average satisfaction scores (all > 4.5 on a 5-point Likert scale), with low standard deviations (0.55–0.71), indicating consistently positive feedback.

From an economic standpoint, the implementation of OPAT with elastomeric pumps at our center between 2019 and 2024 resulted in substantial cost savings compared to exclusive inpatient care. A total of 1,934 OPAT therapy days were delivered, each including daily administration (nursing time and clinic overhead), one initial infectious disease consultation, weekly follow-up visits, and 80 vascular access procedures. The total cost incurred for the OPAT-managed therapy days amounted to 62,190.64 €. In contrast, if these same 1,934 intravenous therapy days had been managed entirely in a hospital setting, the projected cost would have reached 773,600.00 €, based on a standardized daily inpatient rate of 400 €. This corresponds to a net cost saving of 711.409 €, and a relative cost reduction of approximately 92% in favor of the OPAT model. On a per-day basis, inpatient therapy was estimated at 400 € per day, whereas OPAT delivery (including all associated costs) averaged approximately 32.16 € per day. A detailed breakdown of OPAT costs is presented in Table 4.

**Table 4:** OPAT daily cost breakdown and comparison with inpatient care

Cost Item / Category	OPAT	Inpatient Care
Elastomeric pump (per day)	9.96 €	
Nurse time (30 min/day)	11.00 €	
Outpatient clinic overhead (30 min/day)	3.00 €	
Vascular access placement (80 total, prorated)	74.00 € each	
Infectious Disease Consultation - initial (76 total)	29.00 € each	
Infectious Disease follow-up (30 min/weekly)	28.00 €	
**Average cost per day**	**32.16 €**	**400.00 €**
Total treatment days	1,934	1,934
**Total cost**	**62**,**190.64 €**	**773**,**600.00 €**

## Discussion and conclusion

The use of elastomeric pumps in OPAT programs is a cornerstone of these programs, aiming to minimize patient access to hospital services and complications—both infectious and non-infectious—related to hospitalization. This is achieved by using easy-to-use devices that can also be worn at home for medications requiring prolonged infusions. Continuous infusion might simplify care and improve adherence compared with once-daily or multiple daily administrations and – in the case of betalactams – might improve microbiological or clinical efficacy. Unfortunately, not all antimicrobial drugs can be administered via elastomeric pumps, primarily for reasons of chemical, physical, and microbiological stability over the 24 h. For many of these antimicrobials, the problem is not so much the lack of actual stability, but the partial lack of formal data provided by the industry regarding their use via elastomeric pumps.

Our six-year experience demonstrates that elastomeric pump-based OPAT is a safe, effective and economically sustainable alternative to inpatient care for selected patients requiring prolonged intravenous antimicrobial therapy. The high treatment success rate and low recurrence observed in our cohort are consistent with international data, reinforcing the clinical robustness of this strategy. Patient-reported outcomes further confirm the acceptability and tolerability of elastomeric devices in the outpatient setting, which is crucial to adherence and long-term success.

Other studies have reported success rates within a similar range. For instance, Psaltikidis et al. and Voumard et al. each observed cure rates exceeding 95% [1, 15], while Durojaiye et al. reported clinical success between 84% and 88% [2]. Despite differences in geography, patient populations, and antibiotic regimens, these findings collectively highlight the robustness and reproducibility of OPAT outcomes. Our cohort’s treatment success aligns closely with these data, underscoring the reliability of elastomeric pump-based OPAT as an effective therapeutic approach.

Patient selection remains critical to OPAT success. In our cohort, in accordance with international OPAT guidelines [4]– [5, 20], we applied stringent inclusion criteria which likely contributed to the low complication rate. However, daily clinic visits for pump replacement and monitoring may pose challenges for patients with mobility issues or limited caregiver support.

Regarding safety, vascular access complications, primarily thrombosis and device malfunctions, represented the most frequently observed adverse events in our cohort. These findings align with prior literature highlighting catheter-related complications as a major contributor to OPAT-related morbidity. For example, Durojaiye et al. reported that 59% of OPAT complications in a multicenter UK study stemmed from vascular access issues [3]. In Barr et al.’s cohort of over 800 patients, catheter-related problems accounted for a substantial portion of adverse events, though central and midline devices showed no significant difference in infection risk [35]. These data underscore the importance of rigorous catheter management, proper line selection based on treatment duration and patient characteristics, and adherence to best practices and maintenance protocols. Future studies should evaluate strategies to minimize thrombotic complications while ensuring effective antimicrobial delivery.

Moreover, recent research has shown that patients receiving OPAT with aminoglycosides or vancomycin are at a significantly higher risk of readmission due to complications, particularly when therapeutic drug monitoring is insufficient [36]. However, these risks can be mitigated through routine blood tests to ensure optimal dosing and early detection of adverse effects.

From an antimicrobial stewardship perspective, OPAT with elastomeric pumps provides a valuable opportunity to optimize antibiotic use, particularly in infections caused by ESBL-producing organisms. Traditionally, ertapenem has been the preferred outpatient option due to its once-daily dosing. However, continuous infusion of beta-lactam antibiotics via elastomeric pumps offers a carbapenem-sparing approach, enabling the use of alternative agents such as piperacillin/tazobactam or ceftolozane/tazobactam while maintaining optimal pharmacodynamic targets.

Environmental and pharmacotechnical considerations also warrant attention. Skryabina and Dunn emphasized how environmental variables such as ambient temperature, solution viscosity, and external pressure, can affect the flow rate accuracy of elastomeric devices [37]. These fluctuations may lead to under- or overdosing, particularly problematic for time-dependent antibiotics where stable plasma concentrations are critical. Real-world studies, including those by Fernández-Rubio et al. and Esteban-Cartelle et al., have confirmed significant variability in delivery rates and degradation patterns at temperatures exceeding 25–30 °C [24, 25]. Such variability underscores the importance of using validated infusion protocols, temperature shielding, and potentially pharmacokinetic monitoring to maintain consistent drug exposure in ambulatory settings [4, 38].

Similarly, data from the British Society for Antimicrobial Chemotherapy (BSAC) OPAT Drug Stability Testing Programme showed that ceftolozane/tazobactam, though partially stable, exhibited degradation beyond 18 h at 32 °C, especially at higher concentrations.

Therefore, there is a pressing need for a globally harmonized OPAT-specific antimicrobial stability testing framework, integrating real-life data on temperature exposure, concentration limits, degradation profiles, and toxicity risks. This would support clinicians, pharmacists, and stewardship programs in making informed, safe, and sustainable choices, ultimately enabling broader, safer, and more equitable access to continuous infusion therapies across healthcare systems [39].

Patient-reported outcomes from our structured telephone survey confirmed high satisfaction and tolerability, with 83.3% of respondents indicating they would be willing to use the device again. These results reinforce the acceptability of elastomeric pumps in our cohort, a key determinant of adherence and overall success in outpatient care models. Comparable levels of patient satisfaction have been reported in international studies, such as those by Voumard et al., Ferro Rodríguez S et al., Psaltikidis et al. which reported similarly high levels of patient comfort and usability in both supervised and self-administered OPAT settings [1, 15, 40]. These findings suggest that, when accompanied by appropriate education and support, elastomeric infusion systems are well tolerated by patients and compatible with outpatient clinical goals.

A further strength of the pump-based OPAT model lies in its capacity to generate substantial healthcare savings while mitigating patient exposure to hospital-acquired risks. By facilitating early discharge or avoiding hospitalization, OPAT reduces inpatient bed occupancy, alleviates pressure on hospital infrastructure, and may contribute to lower rates of nosocomial infections.

Our cost analysis, though limited to direct hospital expenses, suggests that OPAT with elastomeric pumps offers substantial economic advantages. These findings are broadly in line with previous international studies, although our observed savings are comparatively higher. For instance, Chapman et al. estimated that OPAT services in the UK accounted for 47–62% of the cost of equivalent inpatient care, depending on the clinical setting and model employed [12]. In a Greek cohort, Psaltikidis et al. found that OPAT was 5.5 times less expensive than inpatient intravenous therapy, even when including antibiotic and laboratory expenses [15]. A Turkish prospective study by Bastug et al. further supported the financial sustainability of OPAT, reporting average cost savings of 74%, including transportation and indirect costs [14].

However, direct comparisons across studies should be approached with caution due to methodological heterogeneity. The notably lower per-day cost reported in our analysis reflects a focused cost-minimization strategy, restricted to direct hospital-incurred costs. We did not include expenditures related to antimicrobial agents, routine laboratory monitoring, pre-OPAT hospitalization days, or transportation logistics. Furthermore, indirect societal benefits, such as earlier return to work, reduced caregiver burden, or improved quality of life, were not quantified. Notably, the potential cost savings related to a reduced risk of nosocomial infections, a well-documented benefit of early discharge and outpatient management, were also excluded. Conversely, the economic impact of vascular access-related complications was not factored into the model and could necessitate additional resource use.

Overall, these exclusions render our cost estimates conservative, reinforcing the conclusion that OPAT represents a cost-efficient strategy even under strict hospital accounting frameworks. Future studies incorporating a broader societal perspective and standardized costing methodologies could help define the full economic value of OPAT in diverse health systems.

Despite the study’s strengths, including its real-world setting and comprehensive dataset, some limitations should be acknowledged. The retrospective design may introduce selection bias, and the single-centre nature limits the generalizability of our findings. Longer-term outcomes and prospective multicentric data are needed to better define the sustained impact of OPAT in diverse healthcare settings. Another limitation is the potential recall bias associated in patient-reported outcomes collected via telephone questionnaire, as satisfaction and tolerability perceptions may be influenced by the time elapsed since treatment completion. Unfortunately, systematic collection of questionnaires was only possible starting in 2025, and for this reason some patient responses may be imprecise, introducing a possible bias. Furthermore, only patients who received therapy via elastomeric pump in the in-hospital setting were included, as complete data collection was possible for them. However, many patients, due to mobility limitations or difficulties in accessing the hospital on a daily basis, usually receive OPAT at home. Including these patients could have further strengthened our results, or additional AEs or complication might have been identified if they had been part of the study.

In conclusion, our six-year experience highlights the safety, efficacy, and economic benefits of elastomeric pump-based OPAT, reinforcing its role as a viable alternative to inpatient care for selected patients requiring prolonged antimicrobial therapy.

## Data Availability

No datasets were generated or analysed during the current study.
